# Potential Effects of Metal Oxides on Agricultural Production of Rice: A Mini Review

**DOI:** 10.3390/plants12040778

**Published:** 2023-02-09

**Authors:** Miao Xu, Qi Zhang, Xiuyun Lin, Yuqing Shang, Xiyan Cui, Liquan Guo, Yuanrui Huang, Ming Wu, Kai Song

**Affiliations:** 1 Key Laboratory of Straw Comprehensive Utilization and Black Soil Conservation, Ministry of Education, College of Life Science, Jilin Agricultural University, Changchun 130118, China; 2 Rice Research Institute, Jilin Academy of Agricultural Sciences, Changchun 130118, China; 3 School of Life Science, Changchun Normal University, Changchun 130032, China

**Keywords:** metal oxide nanoparticles/rice, oxidative stress, heavy metal stress, quality improvement

## Abstract

The extensive usage of metal oxide nanoparticles has aided in the spread and accumulation of these nanoparticles in the environment, potentially endangering both human health and the agroecological system. This research describes in detail the hazardous and advantageous impacts of common metal oxide nanomaterials, such as iron oxide, copper oxide, and zinc oxide, on the life cycle of rice. In-depth analyses are conducted on the transport patterns of nanoparticles in rice, the plant’s reaction to stress, the reduction of heavy metal stress, and the improvement of rice quality by metal oxide nanoparticles, all of which are of significant interest in this subject. It is emphasized that from the perspective of advancing the field of nanoagriculture, the next stage of research should focus more on the molecular mechanisms of the effects of metal oxide nanoparticles on rice and the effects of combined use with other biological media. The limitations of the lack of existing studies on the effects of metal oxide nanomaterials on the entire life cycle of rice have been clearly pointed out.

## 1. Introduction

Metal oxide nanomaterials exhibit outstanding physicochemical properties such as a high specific surface area, electron mobility, thermal stability, mechanical strength, and surface defects due to their unique nano-size [1,2,3], allowing for their wide application in adsorbent materials, nano-fertilizers, catalytic materials, nano-pesticides, and pollutant sensors [4,5,6,7,8]. With human activity, nanoparticles are constantly being released into the environment. Once ingested by living things, they might collect in certain tissues or organs and eventually have major impacts [9], leading to new safety hazards in agricultural production [10]. 

Researchers are also very interested in the agroecological toxicological impacts of metal oxide nanoparticles (MONPs). According to earlier research, the main manifestations of MONPs’ harmful effects on plants, which eventually prevent plant growth, are oxidative stress and oxidative damage. Additionally, the type of MONP and plant has a direct bearing on this effect [11]. Furthermore, one of the most significant food crops in the world is rice, a natural producer. In contrast to other crops, rice is grown in a way that promotes the mobility and solubility of metal oxide nanoparticles and increases the impact of these materials [12].

This study examines how various significant MONPs affect rice’s growth and development and discovers that MONPs have dual biological impacts on rice. However, MONPs can also lessen the toxic effects of heavy metals and other toxic substances on rice and increase rice resistance [13,14,15,16], as well as act as nano-fertilizers to increase rice yield and enrich rice seed nutrition [17]. On the one hand, MONPs can inhibit the germination and seedling growth of rice seeds to a certain extent, causing oxidative damage to rice [18,19,20,21]. On the other hand, MONPs can reduce the toxic effects of heavy metals. Additionally, this study provides an overview of previous research, goes into great detail on research-related issues and hot subjects for the following stage, and anticipates difficulties and potential field applications. It is anticipated that it will encourage the green, healthy, and sustainable development of nano-agriculture.

## 2. Effect of MONPs on the Growth of Rice

### 2.1. Iron Oxide Nanoparticles

Iron oxide nanoparticles (Fe_2_O_3_ NPs) are widely utilized in a variety of industries, including catalysis, bioengineering, and medicine, and they gradually enter the agroecological environment mostly through wastewater excretion and atmospheric emissions [22,23]. As a form of nanoparticles, it is inevitable that they have some phytotoxicity toward plants. However, the vast majority of research has demonstrated that Fe_2_O_3_ NPs can help rice seeds germinate, reduce oxidative stress brought on by abiotic stressors, and aid in rice growth. Additionally, they can be applied as a nano-fertilizer to help rice seedlings grow better in unfavorable soil circumstances, including iron deficiency and drought.

**Figure 1:**
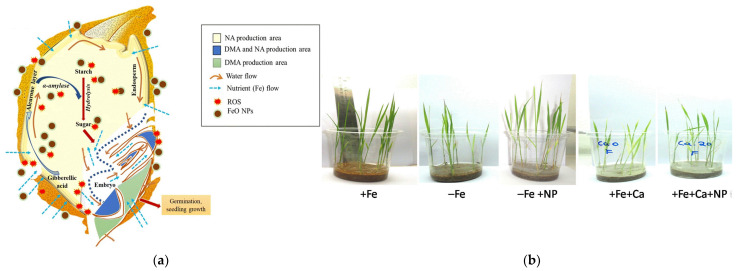
(**a**) A model of iron-oxide-nanoparticle-induced germination in rice. Reprinted with permission from Ref. [24]. 2021, Springer-Verlag; (**b**) rice growth response. Reprinted with permission from Ref. [26]. 2017, American Chemical Society.

As early as 2013, Alidoust et al. demonstrated that citric-acid-coated 6 nm Fe_2_O_3_ NPs can act as an accelerator to increase the length of rice roots and are less toxic than micron iron oxide under reducing conditions [22]. Since then, various iron oxide nanoparticles for rice seed germination have been increasingly studied. Among them, iron oxide nanoparticles prepared from *Cassia occidentalis* L. flower extract were shown to penetrate the rice seed coat [24], inhibit dormancy which enhanced starch metabolism, and significantly promote germination of ecological stress-sensitive, early flowering pure mutant rice (Figure 1a). Fe_2_O_3_ NPs also inhibit the synthesis of growth hormones and abscisic acid in the roots of transgenic and non-transgenic rice [25]. 

In 2017, Sebastian et al. synthesized carbon-encapsulated Fe_3_O_4_ NPs with ferric chloride and caffeic acid to significantly improve calcium-induced Fe deficiency in rice [26] (Figure 1b). This study provided a practical solution to improve Fe deficiency in crops caused by calcareous soils in agriculture. Moreover, Li et al. also found that low doses of zero-valent iron (ZVI) and Fe_3_O_4_ NPs could be used as an alternative to Fe fertilizers and improve plant growth under Fe-deficient conditions by alleviating oxidative stress and regulating phytohormones in rice plants caused by Fe deficiency [23]. In addition, Sainao et al. used MNPs-Fe_3_O_4_ (iron oxide nanoparticles containing both Fe^2+^ and Fe^3+^ ions) to mitigate the toxic effects of 3-nitrophenol on rice seedlings [27,28,29].

**Figure 2:**
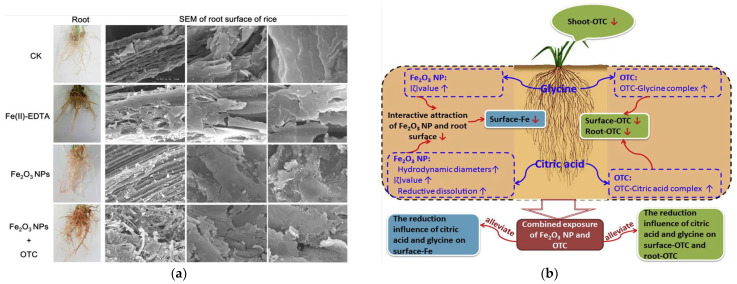
(**a**) Rice root surface images. Reprinted with permission from Ref. [30]. 2019, Springer-Verlag; (**b**) diagram showing that citric acid and glycine reduce the uptake and accumulation of Fe_2_O_3_ nanoparticles and oxytetracycline in rice seedlings upon individual and combined exposure. Reprinted with permission from Ref. [31]. 2019, Elsevier.

The collective impact of nanoparticles and antibiotics on crops in a complex ecological environment is also a hot research topic. Bao et al. treated rice with Fe_2_O_3_ NPs and oxytetracycline (OTC) separately, and their accumulation on the root surface, above-ground parts, and inside the roots showed a decreasing pattern. Meanwhile, the combined treatment increased the distribution of both on the root surface of the rice, where oxytetracycline promoted the adsorption of Fe on the root surface and Fe_2_O_3_ NPs promoted the content of oxytetracycline in the rice roots. This phenomenon may be because OTC stabilizes Fe^2+^ in solution from the reductive dissolution of Fe_2_O_3_ NPs through complexation with Fe^2+^, and Fe_2_O_3_ NPs can eliminate the effect of OTC. This study demonstrates the complexity of the effects of pollution in agroecosystems on rice growth [30] (Figure 2a). Other studies reported that the uptake of Fe_2_O_3_ NPs and oxytetracycline by rice was affected by rice root secretions of citric acid and glycine, which could effectively hinder the bioaccumulation of Fe_2_O_3_ NPs and OTC in rice [31] (Figure 2b).

**Figure 3:**
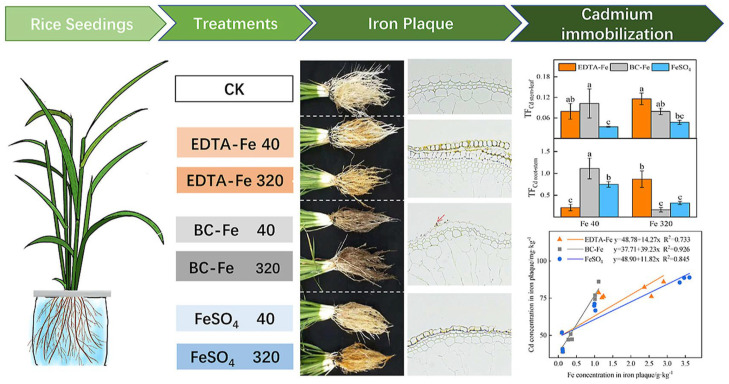
Nano-Fe_3_O_4_-modified biochar promotes Fe film formation and cadmium (Cd) fixation in rice roots. Different letters indicate a significant difference between treatments according to Duncan’s test (*p* < 0.5). Reprinted with permission from Ref. [33]. 2021, Elsevier.

Interestingly, Fe_2_O_3_ NPs can also mitigate heavy metal contamination in rice. In 2019, Sebastian et al. demonstrated the excellent biocompatibility of magnetite nanoparticles prepared using rubber tree bark extract and a mixture of FeCl_3_ and FeCl_2_. Their findings could effectively inhibit the uptake of environmental cadmium (Cd) and sodium by rice, providing a new idea to alleviate food security problems caused by heavy metal pollution in agroecosystems [17]. In the same year, Rizwan et al. found that co-treatment of Fe_2_O_3_ NPs with biochar increased the iron concentration and reduced Cd enrichment in rice seedlings [32]. In addition, nano-Fe_3_O_4_-modified biochar (BC-Fe) treatment also enhanced Cd and Fe sequestration in rice roots and inhibited Cd transport and accumulation in rice tissues [33] (Figure 3). Even in drought-like conditions, Ahmed et al. discovered that co-treating rice with hydrogel nanoparticles and iron oxide nanoparticles prepared from *Bacillus* strain RNT1 was able to reduce the reactive oxygen content and Cd adsorption by rice [34]. Fe_3_O_4_@NH_2_ nanoparticles can be used to immobilize the heavy metal Pb in soil and inhibit Pb uptake by rice roots and shoots [35], and the nanoparticles can be recycled. In 2018, Huang et al. found that Fe_3_O_4_ NPs and zero-valent iron nanoparticles were better at preventing arsenic migration to the above-ground parts of rice seedlings compared to high-quality graphene oxide, multilayer graphene oxide, 20 nm hydroxyapatite (HA_20_), and 40 nm hydroxyapatite (HA_40_) [36]. This study provided a reasonable basis for arsenic pollution management. In 2020, Khan et al. found that Fe_3_O_4_ NPs synthesized by *Bacillus subtilis* significantly inhibited arsenic levels and promoted plant growth at low concentrations [37]. This mechanism can be explained by the fact that Fe_2_O_3_ NPs alleviated oxidative stress in rice, inhibited the enrichment of arsenic in rice roots and leaves, and reduced the toxic effects of arsenic on rice. The nanoparticles also affected the transcription and expression of genes related to iron uptake and transport in rice, which improved iron accumulation in rice roots and leaves under arsenic stress conditions. Eventually, the photosynthetic pigment content of rice and the growth of rice were restored [38]. This research has furthered the development of nanotechnology in the remediation of agricultural land contaminated with heavy metals.

Although the current iron oxide nanoparticle pollution in rice farming is not serious [39,40,41,42], there have only been a few studies compared to other crops, and more thorough investigations are required to broaden the research’s focus and depth.

### 2.2. Copper Oxide Nanoparticles

Due to their excellent thermal, electrical conductivity, catalytic, and antibacterial properties, copper oxide nanoparticles (CuO NPs) are widely used in electronics, chemicals, machinery, and agriculture. These particles also gradually enter the soil and water bodies of the agroecological environment with human activities [12]. The area of agricultural soils that are contaminated with copper is currently growing each year, and the soil’s copper (Cu) concentration is also rising each year, which has varying degrees of negative impacts on crop growth, development, and yield [43]. Copper is a trace element that is involved in numerous metabolic processes in rice. However, excessive copper ions can be hazardous to organisms. Additionally, due to their special characteristics, CuO NPs are more likely to interact with other chemicals [44]. Therefore, there has been a lot of interest in researching the possible effects of CuO NPs on rice growth and development.

Cu’s harmful effects on rice are mostly seen as a reduction in tillering, a delay in fertility, and inhibition of root and shoot growth [45]. CuO NPs typically interact with rice in the form of both the actual nanoparticles and precipitated Cu^2+^, leading to a variety of reactions, such as oxidative stress. Additionally, they benefit rice tissue culture, seed mineral management, and arsenic stress reduction.

**Figure 4:**
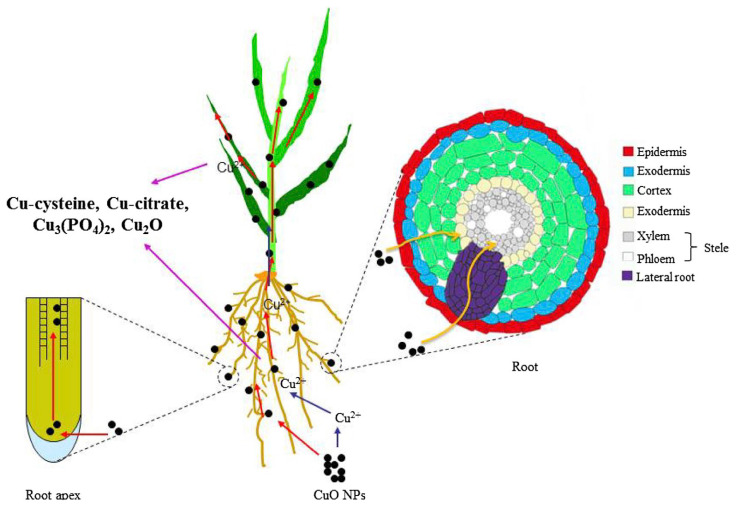
Nano-Fe_3_O_4_-modified biochar promotes Fe film formation and Cd fixation in rice roots. Reprinted with permission from Ref. [12]. 2015, Elsevier.

In 2014, Peng et al. found that CuO NPs could enter the epidermis, ectodermis, and cortex of rice roots under hydroponic conditions, and finally reach the endodermis, but it was difficult to pass through the Casparian strip [12]; however, the formation of lateral roots provided a potential pathway for CuO NPs to enter the stem. During the transfer of CuO NPs, dissolved Cu is bound to cysteine, citrate, salt, and phosphate ligands, in which some Cu(II) is converted to Cu(I). Cu in rice root cells and cell voids exists as Cu-citrate and CuO NPs, respectively [12] (Figure 4). CuO NPs in the environment cause oxidative damage to rice seedlings, which has a negative impact on their growth and development. Rice treated with CuO NPs had significantly lower seed germination and cell viability of seedling roots compared to the control group, as well as a significant build-up of proline and H_2_O_2_ [46,47]. Wang et al. claim that the presence of CuO NPs stimulates the generation of reactive oxygen species (ROS) and copper uptake by rice roots, resulting in oxidative stress in rice, disruption of intracellular metabolism, DNA damage, and changes in the expression of factors that control rice cell cycle processes, ultimately inhibiting rice root growth [13]. Another study found that significant Cu build-up, excessive ROS and lipid peroxidation, modification of antioxidant enzyme activity in shoots and roots, and enhanced lignification were all associated with the toxicity of CuO NPs exposure. The proline and soluble sugar accumulation in the shoots and roots were greatly enhanced by CuO NPs treatment [48]. CuO NPs are primarily concentrated in rice chloroplasts when compared to bulk Cu; nonetheless, this destroys the cystoid membrane, which in turn inhibits rice development and photosynthesis [44,49]. Furthermore, the release of Cu ions from bulk Cu leads to oxidative stress, which in turn hinders rice’s ability to grow and develop [50]. Additionally, Cao et al. tested several combinations of CuO NPs and Sb in its two valence states. They discovered that the number and community structure of rice rhizobacteria on soil nutrient cycling were negatively impacted by the co-exposure of CuO NPs with Sb (III) [51]. These serve as a foundation for additional research on the methods by which nanoparticles move within plants, the effects of ecotoxicology on biochemical parameters, and potential impacts on the food chain [44].

Curiously, the researchers discovered that the interaction between humic acid and CuO NPs enhanced the electrostatic barrier between CuO NPs and rice root cells, reducing the contact between CuO NPs and rice and attenuating oxidative damage to the rice cells. Humic acid can also directly scavenge the reactive oxygen species that CuO NPs create, minimizing their harmful effects on rice [52]. Additionally, it has been demonstrated that Fe^2+^ reduced Cu build-up in rice shoots and roots and hindered copper adsorption in rice roots [53]. Under copper oxide nanoparticle stress, silica and hydrogen sulfide improved the ascorbate–glutathione system and NO concentration in rice (15-day rice seedlings), reducing the oxidative stress caused by rice [54].

**Figure 5:**
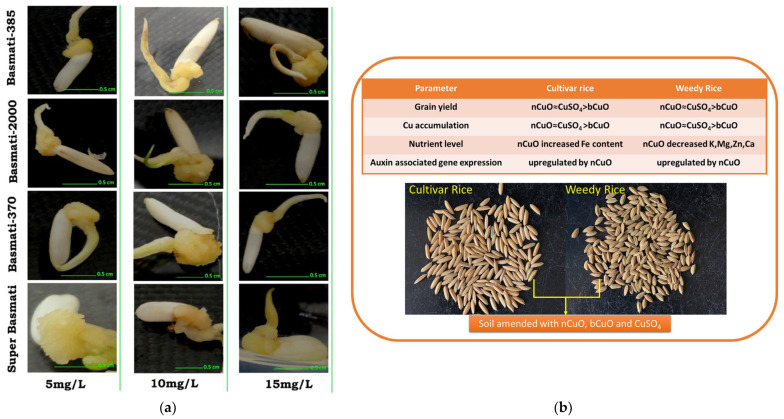
(**a**) Effect of copper oxide nanoparticles (5 mg/L, 10 mg/L, and 15 mg/L) on rice healing tissue production. Reprinted with permission from Ref. [55]. 2016, Frontiers; (**b**) copper oxide nanoparticles affect yield, nutritional quality, and growth-hormone-related gene expression in weedy and cultivated rice (*Oryza sativa* L.) seeds. Reprinted with permission from Ref. [56]. 2022, Elsevier.

As the study progressed, the researchers found that the same beneficial aspects of CuO NPs existed for rice growth. CuO NPs synthesized from *Azadirachta indica* leaf extract showed low toxicity and stable biocompatibility in inducing healing tissue formation in rice [55] (Figure 5a). This research opened up new paths in the field of plant tissue culture. In 2022, Deng et al. examined copper uptake, seed yield and nutritional value, and the expression of auxin-associated genes in weedy and cultivated rice. They found that CuO NPs not only promoted the expression of auxin-associated genes in these two rice species but also facilitated the enhancement of iron content in cultivated rice seeds [56] (Figure 5b). This study provided valuable information for the delivery system of nano-fertilizers or crop protection materials.

**Figure 6:**
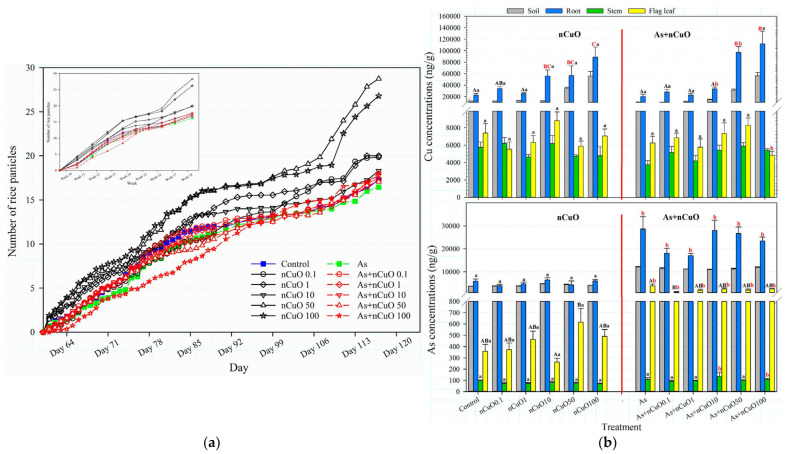
(**a**) Heading process of rice panicles after 131 days of exposure to arsenic in soil and copper oxide nanoparticles in the nutrient solution. Reprinted with permission from Ref. [57]. 2018, American Chemical Society; (**b**) copper and arsenic concentrations in the mature rice plants from a greenhouse study of rice (*O*. *sativa japonica* ‘Koshihikari’) with 131-day exposure to arsenic in soil and copper oxide nanoparticles in the nutrient solution. Means of treatments at the same As level with a common superscript letter (A–C) are similar (*p* < 0.05). Means of treatments at the same CuO NPs level with a common superscript (a and b) letter are similar (*p* < 0.05). Reprinted with permission from Ref. [58]. 2019, American Chemical Society.

Additionally, CuO NPs, like iron oxide nanoparticles, inhibit arsenic uptake while attenuating the detrimental effects of arsenic stress on rice shoot length and root branching number [18]. Stress treatment with arsenic and CuO NPs alone significantly reduced the rice germination rate, especially inhibiting the growth of the above- and below-ground parts of seedlings. However, when the two nanoparticles were applied to the rice, CuO NPs shortened the rice tassel stage, accelerated rice maturation, and reduced the arsenic content in rice seeds [57,58] (Figure 6). Consistent with this study, Wang et al. found that CuO bulk particles, CuO NPs, and Cu^2+^ could reduce the amount of arsenic (III) in the seeds in total arsenic throughout the life cycle of rice [59].

At the physiological level of the plant, CuO NPs are known to have two effects on the growth of rice seedlings. The dominant negative effects of CuO NPs are mainly a reduction in the germination rate of rice, decreased photosynthetic efficiency, induced oxidative stress in rice, and in more severe cases, the death of rice seedlings. On the other hand, CuO NPs also play a vital role in rice tissue culture, the regulation of minerals in seeds, and the improvement of land pollution caused by arsenic. The following work should explain the adsorption capacity of CuO NPs at various times and look into the transport mechanism of CuO NPs in rice in more detail. In order to achieve the best bacterial suppression while minimizing toxicity, the ideal dose of this nanomaterial in plant tissue culture was investigated. Research is being carried out on the molecular mechanisms of CuO NPs in rice seed mineral control and arsenic stress reduction.

### 2.3. Zinc Oxide Nanoparticles

Zinc oxide nanoparticles (ZnO NPs) are one of the widely utilized MONPs, showing promising applications in medicine, textiles, sensors, optical materials, catalysts, optical materials, and ceramics [60]. As human activities continue to expand, this nanomaterial continues to flow into nature. Investigating the effects of ZnO NPs on the growth of rice, a globally important crop, is of interest to the community. 

**Figure 7:**
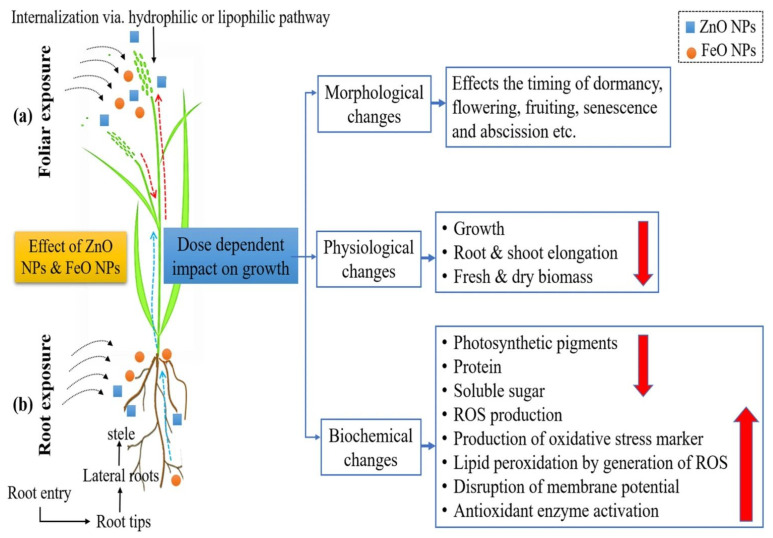
Schematic representation of the dose-dependent effects of ZnO and FeO NPs on morphological, physiological, and biochemical changes in rice crops, (**a**) foliar exposure of NPs via. lipophilic pathway; (**b**) uptake of NPs from soil in the root system. Reprinted with permission from Ref. [64]. 2021, Springer-Verlag.

Previous studies have shown that ZnO NPs can be absorbed by the roots of rice seedlings, causing stomatal closure and damage to the ultrastructure, accelerating the synthesis of the phytohormone ethylene, causing oxidative stress in rice seedlings, and significantly inhibiting the growth of rice seedling roots [14,61,62]. However, rice *FT-INTERACTING PROTEIN 7* enhances rice tolerance to ZnO NPs by inhibiting auxin synthesis [60], while the exogenous application of melatonin alleviates the oxidative damage induced by ZnO NPs and abates the inhibitory effect on rice growth [63]. In addition, ZnO NPs synthesized using *Senna occidentalis* L. leaf extract acted on rice by root exposure and foliar spraying [64], reducing photosynthetic efficiency and affecting dormancy time, flowering, and fruit set in rice (Figure 7). However, seed priming with polyethylene glycol will slightly mitigate this toxic effect [65].

**Figure 8:**
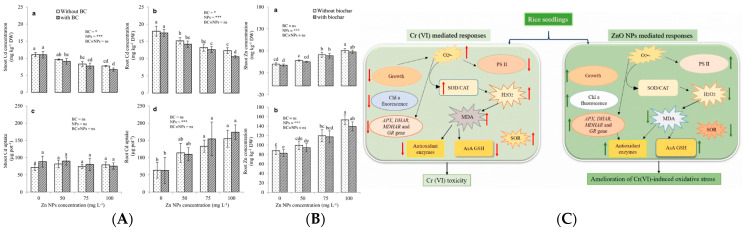
(**A**) Concentrations and total uptake of Cd in the shoots and roots of rice treated with biochar and ZnO NPs. (a) Cd concentrations in shoots; (b) Cd concentrations in shoots; (c) Cd uptake by shoots; (d) Cd uptake by roots. Values are means of four replications and bars represent standard deviation. Different letters demonstrate significant differences among treatments. In figures, ns = non-significant; * = significant at 0.05, and *** = significant at 0.001 levels; (**B**) Concentrations of Zn in the shoots and roots of rice treated with biochar and ZnO NPs. (a) Zn concentrations in shoots; (b) Zn concentrations in shoots. Values are means of four replications and bars represent standard deviation. Different letters demonstrate significant differences among treatments. In figures, ns = non-significant; * = significant at 0.05, and *** = significant at 0.001 levels. Reprinted with permission from Ref. [66]. 2019, Springer-Verlag; (**C**) probable model for Cr(VI)-induced stress and ZnO NPs’ action in response to Cr(VI) toxicity in rice seedlings. Reprinted with permission from Ref. [70]. 2022, Elsevier.

ZnO NPs are crucial in reducing Cd stress in rice because Cd and zinc (Zn) are environmental competitors, share many chemical characteristics, and have the same uptake pathways in plants. In 2019, Zhang et al. reported that ZnO NPs could enhance soil pH and reduce the toxic effect of Cd on rice. The higher concentration had the most significant promotion effect on the early growth of rice, as demonstrated by increasing the biomass, tiller number, and plant height of rice [19]. In the same year, Ali et al. used foliar sprays to increase rice biomass and photosynthesis using only ZnO NPs or combined with biochar, with the latter effectively reducing the Cd concentrations in rice roots and increasing Zn concentrations in rice rhizomes [66] (Figure 8A,B). Similar phenomena were observed by Faizan, Li, and Wang et al. [67,68,69]. ZnO NPs also improved the growth and photosynthetic efficiency limit of rice seedlings under chromium and arsenic stress [70,71] (Figure 8C). Arsenic uptake by rice seedlings was reduced by increasing the rice biomass and Zn content. Foliar sprays of ZnO NPs in the presence of salicylic acid alleviated arsenic-induced oxidative stress in rice through transient excitation of the antioxidant system [72]. In addition, Akhtar et al. found that ZnO NPs could reduce the gene expression induced by heavy metal stress in rice with the assistance of bacteria (*Bacillus cereus* and *Lysinibacillus macrolides*) while increasing nitrogen content and protein expression, enhancing tolerance, and reducing heavy metal (especially Cu and Pb) toxicity [73,74].

**Figure 9:**
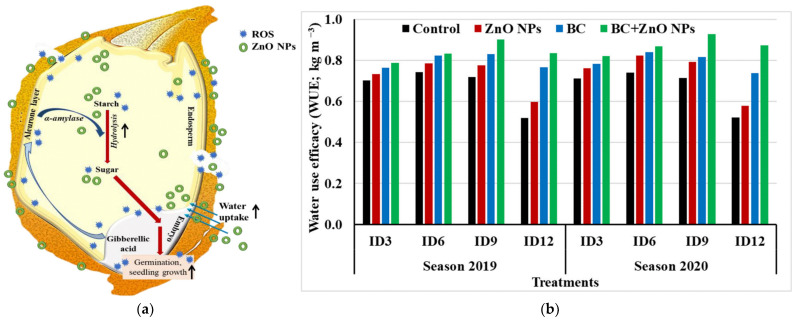
(**a**) Expected mechanism of ZnO-nanoparticle-induced germination in rice seeds. Reprinted with permission from Ref. [77]. 2021, Elsevier; (**b**) interaction effects between irrigation deficit and applications of biochar, ZnO NPs, and their combination treatments on water use efficacy (WUE) during the 2019 and 2020 seasons. Reprinted with permission from Ref. [79]. 2022, Multidisciplinary Digital Publishing Institute.

Furthermore, ZnO NPs play a significant additional role as a Zn supplement for crops. In 2019, Bala et al. implemented foliar spraying of ZnO NPs on rice grown in Zn-deficient soils and found an increase in Zn content in the seeds, which provides theoretical support for the positive application of nanoparticles in crop fertilizers [75]. In the same year, Itroutwar et al. showed positive effects on seedling parameters such as germination, shoot length, root length, and leaf length of rice seeds using bio-derived ZnO NPs synthesized from *Turbinaria ornata* extract [76]. When ZnO NPs prepared from brown algae are applied to rice in combination with a conventional Zn sulfate fertilizer, high-yielding and more nutritious (N, K, Zn) rice was obtained [21]. In 2021, Sharma et al. used ZnO NPs prepared from a *Senna occidentalis* leaf as a nano-initiator to pre-treat seeds of an early flowering pure mutant to improve rice seed germination, seedling vigor, and zinc content in seedlings [77] (Figure 9a). Adhikary et al. adopted the idea and used ZnO NPs and selenium as initiators for rice seeds [78], which enhanced seed vigor and improved conditions such as poor seedling emergence triggered by direct sowing practiced for rice cultivation in South Asia. In addition, Elshayb et al. sprayed ZnO NPs on rice with biochar (BC) as a soil amendment. They found that this approach could mitigate the adverse effects of rice growth due to water deficiency and improve water use efficiency [79] (Figure 9b) holds promise for achieving the goal of increasing food production in arid regions.

The effect of ZnO NPs on rice is greater on Zn^2+^, and the mechanism of its induced changes in rice physiological levels and interactions with molecules such as polyethylene glycol has not been given a comprehensive explanation. Future studies could focus on the biotransformation of ZnO NPs occurring in rice and the plant responses induced by the co-occurrence effects with other media in the rice field such as the inter-competitive effects of Cd^2+^. This provides a theoretical basis for improving food quality, safety issues, and agricultural yields.

### 2.4. Other Metal Oxide Nanoparticles

Cerium dioxide nanoparticles (CeO_2_ NPs) have the unique electronic layer structure of rare earth elements and strong redox ability and are often used in biomedical antioxidants, automotive catalysts, UV-absorbing materials, and antimicrobial functional materials [80]. This nanomaterial gradually flows into the environment with the emission of exhaust gases and vehicle exhaust is absorbed by rice through the root system and stomata, etc., and affects the growth and development of rice [81].

**Figure 10:**
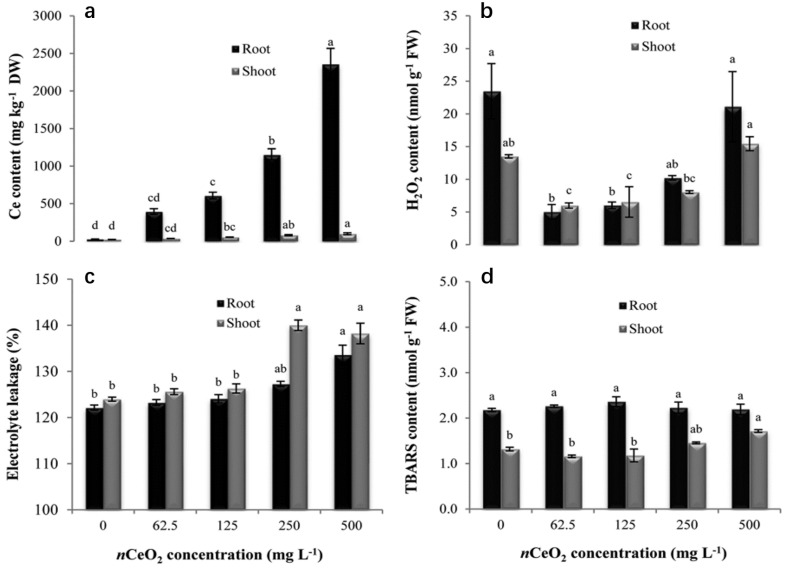
Cerium concentration (**a**), H_2_O_2_ generation (**b**), electrolyte leakage (**c**), and TBARS contents (**d**) in seedling tissues of a high amylose rice variety germinated and grown in nCeO_2_ suspensions for 10 days. Means with the same letter are not significantly different at Tukey’s test (*p* ≤ 0.05). Reprinted with permission from Ref. [83]. 2021, American Chemical Society.

The effects of CeO_2_ NPs on oxidative stress, membrane damage, antioxidant enzyme activity, and macromolecular changes in the roots of rice seedlings were investigated by Rico et al. in 2013 [82]. They noted that the cerium content in rice positively correlated with the concentration of nanoparticles, but the impacts on rice seedlings were insignificant [83] (Figure 10). The team analyzed the effects of CeO_2_ NPs on cerium (Ce) accumulation, antioxidant properties, and nutrient composition in three rice varieties with high, medium, and low straight-chain starch, and found that CeO_2_ NPs were able to reduce the content of iron, proline, and starch in rice grains, and reduce all of the antioxidant values in the grains except for flavonoids. Rice of medium straight-chain starch varieties was the most sensitive to CeO_2_ NPs [84]. Rico et al. later found that CeO_2_ NPs could promote protein synthesis and change the carbohydrate composition in the xylem of rice roots [85]. Citric acid secreted by rice roots helps CeO_2_ NPs to accomplish more cerium accumulation in rice, while the iron film formed on the root surface of rice in an iron-rich environment can reduce the cerium content in rice in the presence of citric acid [81]. Additionally, CeO_2_ NPs with a polyacrylic acid coating could regulate the expression of nitrate reductase genes, promote NO synthesis, and ultimately enhance the salt tolerance of rice [86]. This study enhanced the salt tolerance and yield of rice without increasing the cerium content in rice seeds, opening a new pathway to improve the yield and resistance of saline crops.

**Figure 11:**
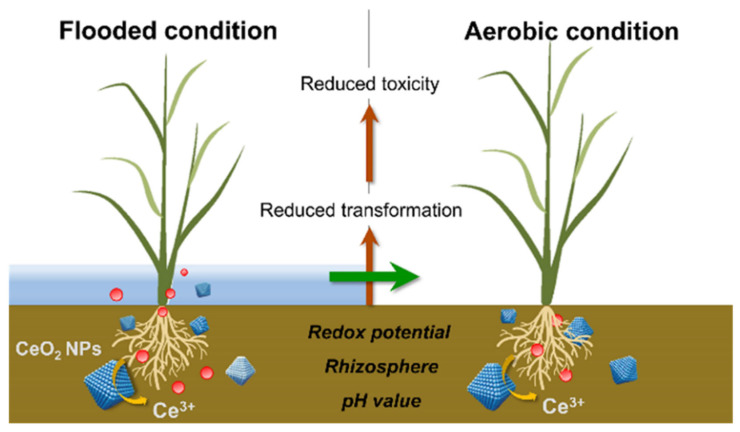
Nano-Fe_3_O_4_-modified biochar promotes Fe film formation and Cd fixation in rice roots. Reprinted with permission from Ref. [80]. 2021, American Chemical Society.

As research continues, researchers believe that the biological effects of CeO_2_ NPs on rice may be biphasic. Zhang et al. found that CeO_2_ NPs had a ‘low promotion and high inhibition’ effect on the growth and development of rice when grown on flooded soil and aerobic soil, with a more pronounced effect in rice grown on flooded soil. Rice grown in flooded soil had higher cerium levels in its shoots. This phenomenon may be due to the soil’s lower pH and redox potential due to its contact with water, which affects the accumulation and transformation of CeO_2_ NPs in rice [80] (Figure 11). It has also been shown that under hydroponic conditions, CeO_2_ NPs alleviate oxidative damage in rice due to nitrogen stress [15]. In contrast, when the nitrogen supply is normal, CeO_2_ NPs trigger oxidative stress and inhibit the normal growth of rice. In 2020, Peng et al. explored the bioavailability and transport of three types of nanoparticles in soil and rice, i.e., zinc oxide, copper oxide, and cerium oxide. The MONPs altered the soil properties while the effects on plant growth were inextricably linked to the type and solubility of the MONPs. This work was crucial for assessing the environmental risk of MONPs in soil and ensuring the safety of agricultural products [87].

Titanium dioxide nanoparticles are used in fertilizers, coatings, plastics, pesticides, cosmetics, etc. [88]. These applications provide opportunities for the influx of titanium dioxide nanoparticles into agroecosystems. It is critical to understand the impact of this nanomaterial on rice growth. 

**Figure 12:**
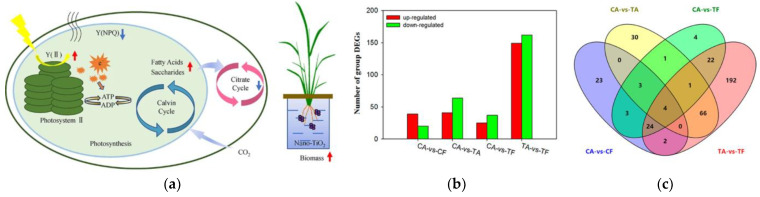
(**a**) Photosynthesis and related metabolic mechanisms of TiO_2_ nanoparticles for rice growth. Reprinted with permission from Ref. [88]. 2020, Springer-Verlag; (**b**) number of differentially expressed genes (DEGs) between each group; (**c**) Venn diagram showing the number of significant DEGs in each group. CA, CF, TA, and TF indicate ambient CO_2_ control, elevated CO_2_ control, ambient CO_2_ with nano-TiO_2_, and elevated CO_2_ with nano-TiO_2_, respectively. Reprinted with permission from Ref. [97]. 2019, American Chemical Society.

Studies have shown that TiO_2_ NPs can reduce rice biomass, enhance antioxidant system defense, and interfere with rice metabolism [89]. By improving energy storage in photosynthesis and decreasing energy waste in rice metabolism, TiO_2_ NPs can improve rice growth and increase rice yield [88]. (Figure 12a). Further investigation revealed that 750 mg/kg of TiO_2_ NPs decreased rice growth and enzyme activity in the soil, but 500 mg/kg of TiO_2_ NPs co-treated with 20 mg/kg of phosphorus or pulverized clay promoted rice growth and development [90,91]. These papers offer important information about the potential uses and dangers of titanium dioxide nanoparticles in agricultural productivity.

TiO_2_ NPs are similar to the previously mentioned MONPs and can also alleviate the toxic effects of Cd and arsenic on rice [20,92,93,94]. Moreover, the combined action of TiO_2_ and CeO_2_ nanoparticles and humic acid can reduce the adsorption of Cu to seedlings and alleviate the toxic effect of Cu on seedlings [95]. In contrast, its co-treatment with tetracycline on rice seedlings leads to severe iron deficiency in rice as tetracycline increases the accumulation of titanium in rice, while TiO_2_ NPs inhibit the adsorption of tetracycline to rice and alleviate the toxic effect of tetracycline on rice [96]. These studies have focused on the potential effects of nanoparticles on crops under conditions of coexistence with other environmental pollutants, facilitating future remediation of complex environments. Based on earlier research, Du et al. observed rice throughout its entire life cycle and found that elevated CO_2_ concentrations could encourage rice growth when TiO_2_ NPs were present [97] (Figure 12b,c) and that an increase in CO_2_ would alter the nutrient value of TiO_2_ NPs for rice and the function of the soil microbial community [98]. The team’s findings provide new ideas on the tolerance of rice to climate and environmental changes.

In addition, α-MoO_3_ nanoparticles also have toxic effects on rice seedlings, leading to oxidative stress in rice [16]. High concentrations of Y_2_O_3_ nanoparticles not only inhibit rice germination and root growth, but also cause oxidative damage to rice cells. However, low concentrations of Y_2_O_3_ nanoparticles can promote the growth and development of rice seedling roots [99]. In addition, Ahmed et al. synthesized magnesium oxide nanoparticles to alleviate the stressful effects of arsenic on rice using natural enterobacteria. The nanoparticles could significantly inhibit the uptake of arsenic in rice, promote the growth of rice under arsenic stress, and reduce oxidative damage in rice [100].

In summary, CeO_2_ NPs and TiO_2_ NPs have a dual effect on rice, while both help to ameliorate the stress on rice growth by other environmental pollutants in the environment. It is noteworthy that two nanomaterials should be the next topic of focus in enhancing the tolerance of rice facing harsh climatic and environmental changes. The effects of MONPs other than these two on rice growth and development have only been reported sporadically. There are still many gaps in the mechanisms related to the effects of nanomaterials on rice growth and development, and future studies should clarify their accumulation, transport mechanisms, and biotransformation within rice at different times, focusing on the potential effects of the combined effects of these nanomaterials and complex factors in the environment on rice growth and development.

Considering the dual effects of MONPs on rice growth and development, the important indicators are summarized (Table 1 and Table 2).

## 3. Conclusions and Outlook

Studies conducted in the past have demonstrated that all of the effects of metal oxide nanoparticles on rice development and growth have a two-fold biological effect. Even though the results of the current study indicate that iron oxide nanoparticles have no discernible toxic effects on rice, further research is needed to determine how to prevent and control the contamination of rice farmland with iron oxide nanoparticles in order to take the necessary precautions. There have been few investigations on the phytotoxicity of other metal oxide nanoparticles on rice, and the deeper molecular causes are still unclear. In order to maximize the benefits of nanoparticles in promoting sustainable agriculture while minimizing the ecological threat of nanomaterials and managing environmental risks, high-throughput fast detection systems must be developed.

The findings of the study on the phytotoxicity of conventional nanoparticles can be used to inform the investigation of current metal oxide nanomaterials on rice growth and development. For instance, the reaction to oxidative stress, the precipitation of metal ions, the application method, the exposure level, etc. In order to increase agricultural rice production, special consideration should be given to the impacts of metal oxide nanoparticles on rice throughout its life cycle. Due to the complexity of the agroecological environment, research should not only focus on one metal oxide nanomaterial’s effect on rice growth but also on the combined effect of nanomaterials and other media in the ecosystem on rice growth. It can also examine how rice responds behaviorally to essential elements, how metal oxide nanomaterials transform, how much rice is produced, and the quality of the seeds.

More importantly, nano-fertilizers have a large market for MONPs. The use of MONPs should be rigorously regulated in terms of timing and frequency, and their content should undergo frequent testing. Avoid secondary harm to rice as much as you can from improper application and excessive concentration. Additionally, the real environmental release of metal oxide nanoparticles, their environmental stability, and the combined effects of wind, heat, rain, and drought must all be taken into account when applying the theoretical advice of the laboratory findings to on-farm production. This offers a workable way to enhance the growth, quality, and yield of rice in agricultural lands that are dry, salty, and excessively polluted.

## Figures and Tables

**Table 1:** Toxic effects of metal oxide nanoparticles on rice growth.

Types of Metal Oxide Nanoparticles	Particle Size	Concentration	Exposure Time	Effects	References
**Copper oxide nanoparticles**	<50 nm	62.5, 125, and 250 mg/L	7 days	It causes oxidative damage to rice, reduces the synthesis of chlorophyll and carotenoids, and inhibits the growth of rice seedlings.	[46]
	<50 nm	0.5 mM, 1.0 mM, and 1.5 mM	14 days	It inhibited rice germination and root vigor, reduced carotenoid content, and increased rice proline, malondialdehyde, and hydrogen peroxide.	[47]
	<50 nm	5 mg/L	3 days	It disrupted rice cell metabolism, DNA damage, and inhibition of *OsCDC2* and *OsCYCD* expression in rice roots.	[13]
	40 nm	10, 50, 100, 500, 1000, and 2000 mg/L	7 days	Increased Cu, prolineand soluble sugar content in rice rhizomes to inhibit seed germinationand early seedling growth.	[48]
	<50 nm	2.5, 10, 50, 100, and 1000 mg/L	30 days	It promotes Cu uptake, induces oxidative stress, and inhibits germination rate, photosynthesis, and root and stem elongation in rice.	[44]
	<50 nm	1, 5, 10, 20, 30, 40, 50, and 100 mg/L	120 days	Inhibiting photosynthesis, decreasing ascorbic acid content, increasing H_2_O_2_, malondialdehyde content and antioxidant enzyme activities, CuO NPs above 50 mg/L produced oxidative damage to rice plants.	[49]
	<50 nm	2.5, 10, 50, 100, and 1000 mg/L	30 days	CuO NPs accumulate in chloroplasts, leading to delamination and deformation of the cystoid membrane.	[50]
**Zinc oxide nanoparticles**	<50 nm	25, 50, and 100 mg/L	7 days	It causes oxidative damage to rice and reduces rice seedling biomass and chlorophyll content to inhibit the growth of rice seedlings.	[14]
	<5 nm	2000 mg/L	7 days	Inhibition of rice root elongation.	[61]
	30 nm	100, 250, 500, and 750 mg/L	7 days	Reduced chlorophyll content of rice seedlings, induced stomatal closure and ultrastructural damage through oxidative stress, and induced ethylene biosynthesis in rice seedlings.	[62]
	37 ± 2 nm	10, 50, 100, and 500 mg/L	Hourly treatment for 10 h, 4 weeks	It inhibits the elongation of rice rootstocks and reduces dry and fresh weight and photosynthetic efficiency.	[64]
**Cerium Oxide Nanoparticles**	8 ± 1 nm	62.5, 125, 250, and 500 mg/L	10 days	It inhibits antioxidant enzyme activity and causes membrane damage.	[82]
	8 ± 1 nm	62.5, 125, 250, and 500 mg/L	10 days	High concentrations caused enhanced electrolyte leakage and lipid peroxidation in seedlings.	[83]
	8 ±1 nm	500 mg/kg	135 days	Reduces the content of iron, proline and starch in rice grains, and reduces all antioxidant values in grains except for flavonoids.	[84]
	<25 nm	500 mg/kg	28 days	Decrease protein and soluble sugar content in the root system, inhibit the uptake and accumulation of macro trace elements in rice seedlings, etc.	[80]
	23.5 ± 6.7 nm	100 and 500 mg/L	3 weeks	The presence of cerium oxide nanoparticles triggers oxidative stress and inhibits average growth in rice when Nitrogen supply is normal.	[15]
**Titanium dioxide nanoparticles**	293 ± 17 nm	100, 250, and 500 mg/L	14 days	Reducing the biomass of rice, enhancing the antioxidant system’s defense and interfering with rice’s metabolism.	[89]
	26.5 nm	500 and 750 mg/kg	After 15 days of incubation to nutrition stage	750 mg/kg exhibited toxic effects of reduced biomass, increased H_2_O_2_ production, lipid peroxidationand electrolyte leakage.	[90]
**Molybdenum oxide nanoparticles**	21.34 nm	100, 500, and 1000 ppm	10 days	Reduced rice photosynthetic pigment levels and caused oxidative stress in rice.	[16]
**Yttrium oxide nanoparticles**	20–30 nm	1, 5, 10, 20, and 50 mg/L	7 days	High concentrations inhibit rice germination and root growth and cause oxidative damage to rice cells.	[99]

**Table 2:** Positive effects of metal oxide nanoparticles on rice growth.

Types of Metal Oxide Nanoparticles	Particle Size	Concentration	Exposure Time	Effects	References
**Iron oxide nanoparticles**	6 nm	500, 1000, and 2000 mg/L	14 weeks	Promote the growth of the rice root system.	[22]
	28 nm	20 and 40 mg/L	24 h	Increase α-amylase activity, promote starch decomposition, and improve rice seed germination rate and seedling vigor.	[24]
	<10 nm	20 mg/L	21 days	Under calcium stress, the nanoparticles enhanced bioproductivity, photosynthetic electron transport, antioxidant enzyme activity, and iron accumulation.	[26]
	20–30 nm	50, 250, and 500 mg/L	2 weeks	Alleviating oxidative stress in rice improves plant growth under iron deficiency conditions and regulates iron-deficiency-induced phytohormones.	[23]
	14.1 nm	2000 mg/L	5 days	Promote the growth, reactive oxygen species production, antioxidant enzyme activity, and chlorophyll content of rice seedlings. Alleviate the physiological toxicity of 3-nitrophenol to rice seedlings.	[29]
	10–50 nm	0.0025 mg/kg	40 days	Increasing chlorophyll and potassium content helps to alleviate oxidative stress under cadmium (Cd) and sodium stress.	[17]
	50–100 nm	10, 20, and 30 mg/L	3 weeks	Increasing rice biomass and iron concentration in rice reduces the enrichment of Cd in rice.	[32]
	18–94 nm	25, 50, and 100 mg/kg	30 days	They improve rice plant biomass, antioxidant enzyme content, and photosynthetic efficiency, reduces reactive oxygen species, and alleviates Cd and drought stress.	[34]
	NA *	40 and 320 mg/L	6 days	Increase in dry weight of rice and transport and accumulation of Cd in rice tissues.	[33]
	5–10 nm	125 mg/kg	15 days	Reduce the concentration of lead in rice roots and shoots.	[35]
	21.3 nm	200 mg/L	5 days	Better performance in preventing the transport of arsenic (As) to the above-ground parts of rice seedlings.	[36]
	60–80 nm	5, 10, and 15 ppm	5 days	They inhibit the uptake of As in rice and promote plant growth.	[37]
	20–30 nm	25 and 50 mg/L	21 days	They improve iron uptake and resistance to oxidative stress in rice and reduce As accumulation in rice.	[38]
**Copper Oxide Nanoparticles**	40 ± 5 nm	1–20 mg/L	10–12 weeks	They have a good effect in inducing rice healing tissue formation.	[55]
	10–100 nm	75, 150, 300, and 600 mg/kg	4 months	Increased iron content and expression of growth-hormone-related genes in cultivated rice seeds.	[56]
	NA *	0.1, 1, 10, 50, and 100 mg/L	18 days	Mitigating the adverse effects of As stress on rice shoot length and root branch number, and reducing As uptake by rice.	[18]
	23–37 nm	0.1–100 mg/L	131 days	The accelerated tassel stage helps shorten rice’s life cycle, thus reducing the accumulation of As in the seeds.	[57]
	9–22 nm	100 mg/kg	104 days	Mitigate the phytotoxicity of As, improve rice yield, and alter the accumulation of As in rice tissues.	[59]
**Zinc oxide nanoparticles**	30–40 nm	50, 100, and 500 mg,/kg	4 months	The higher the concentration, the more significant the promotion effect on the early growth of rice, which can increase the biomass, tiller number, and plant height of rice.	[19]
	20–30 nm	50, 75, and 100 mg/L	Spray on the 14th, 21st, 28th, and 35th day after transplanting	Increasing the biomass and photosynthesis of rice plants significantly reduced the concentration of Cd in seedlings and roots and increased the concentration of Zn in seedlings and roots.	[66]
	11–21 nm	50 mg/L	5 days	Increasing rice biomass, photosynthesis, protein, antioxidant enzyme activity, mineral nutrient content and reducing Cd levels all had significant effects.	[67]
	30 ± 10 nm	25, 50, and 100 mg/L	20 h	Promoting the growth of rice seedlings under Cd stress.	[68]
	15–137 nm	100 mg/L	6 days	Promoting the growth of rice seedlings under As stress and inhibiting As uptake by rice.	[69]
	<100 nm	25 μM	7 days	Reduce the toxicity of chromium (Cr) to rice and promote the growth of rice seedlings.	[70]
	20–30 nm	10, 20, 50, 100, and 200 mg/L	12 days	They promoted rice germination, increased rice biomass and Zn content, and inhibited the accumulation of As in rice.	[71]
	30 nm	1000 mg/L	7 days	Mitigation of oxidative stress induced by As stress in rice.	[72]
	30–70 nm	5, 10, 15, 20, and 25 mg/L	7 days	Increase the tolerance index of rice and reduce the toxic effects of Pb and Cu on rice.	[73]
	30–50 nm	5 and 10 mg/L	21 days	Reduced stress-induced gene expression and increased nitrogen protein content and protein expression in rice.	[74]
	50–70 nm	0, 0.5, 1, and 5 g/L	60 days (every 15 days)	Increase the zinc content in rice and reduce the symptoms of zinc deficiency in rice.	[75]
	14.95 nm	5, 10, 25, 50, 100, and 200 mg/L	12 h	Significantly improved the rice germination rate, increased stem and root length and seedling vigor, etc.	[76]
	31.4 nm	20, 40, and 60 mg/L	4 days	Improved rice yield and enriched rice seed nutrition.	[21]
	40–100 nm	20 mg/L	24 h	Promote rice germination and increase antioxidant enzyme activity, seedling length, and fresh weight, etc.	[77]
	<10 nm	10 μmol	7 days	Increase chlorophyll, phenol and protein content, leaf area index, growth rate, and rice yield.	[78]
	48.70 nm	50 mg/L	Irrigation every 3, 6, 9, and 12 days	Physiological traits such as chlorophyll content, relative water content, plant height, leaf area index, and yield-related components were significantly increased.	[79]
**Cerium Oxide Nanoparticles**	231 ± 16 nm	≥125 mg/L	4 days	Promoting protein synthesis in rice roots and changing carbohydrate composition in the xylem of rice roots.	[85]
	5.6 ± 0.2 nm	98 μg/L and 0.98 mg/L	8 days and 2 months	Regulation of nitrate reductase gene expression to promote NO synthesis and ultimately enhance salt tolerance in rice.	[86]
	23.5 ± 6.7 nm	100 and 500 mg/L	3 weeks	Mitigation of oxidative damage in rice due to nitrogen stress.	[15]
**Titanium dioxide nanoparticle**	5–10 nm	0.1, 1, 10, and 100 mg/L	21 days	Increase energy storage in photosynthesis, reduce energy loss in rice metabolism, realize the promotion of rice growth, and increase rice yield.	[88]
	32.7 nm	500 mg/kg	10 weeks	Increasing chlorophyll content and stem and root length in rice.	[91]
	18–166 nm	100 and 1000 mg/L	10 days	Improved photosynthetic efficiency and chlorophyll content and reduced Cd uptake and distribution in rice roots and leaves.	[20]
	60 ± 11 nm	5, 10, 20, and 30 mg/L	4 weeks (once every week)	Increasing chlorophyll content and rice biomass and reducing Cd uptake in rice.	[92]
	NA *	10, 100, and 1000 mg/L	7 days	Reduced As uptake and oxidative stress in rice.	[93]
	20–40 nm	50, 100, and 500 mg/kg	3 months	They significantly increased plant height and total chlorophyll content at the tillering stage, reduced malondialdehyde content at the gestation stage, and reduced hydrogen peroxide content at the tasseling stage.	[94]
	<20 nm	100 and 1000 mg/L	3 days	Reduce the adsorption of Cu to seedlings and alleviate the toxic effect of Cu on seedlings.	[95]
	10–25 nm	500, 1000, and 2000 mg/L	10 days	Inhibited the adsorption of tetracycline to rice and alleviated the toxic effect of tetracycline on rice.	[96]
	< 100 nm	200 mg/kg	132 days	Under the condition of elevated CO_2_ concentration, they increased rice plant height, stem biomass, and spike biomass and promoted rice growth.	[97]
	20–100 nm	50 and 200 mg/kg	6 months	Under the condition of elevated CO_2_ concentration, they can promote the growth of rice and increase the content of calcium, magnesium, manganese, phosphorus, zinc, and titanium.	[98]
**Molybdenum oxide nanoparticles**	20–30 nm	1, 5, and 10 mg/L	4 h and 15 days	Promote the growth and development of rice seedling roots.	[99]
**Yttrium oxide nanoparticles**	38–57 nm	200 mg/kg	30 days	Significantly inhibit the uptake of As in rice, promote the growth of rice under As stress, and reduce oxidative damage in rice.	[100]

* Note: “NA” in the table means the related information is not provided or available.

## Data Availability

Not applicable.
